# Role of residual mandibular teeth after computer-assisted mandibular reconstruction using a fibular flap

**DOI:** 10.1016/j.jpra.2024.10.002

**Published:** 2024-10-16

**Authors:** Koreyuki Kurosawa, Akira Ohkoshi, Ryo Ishii, Naoko Sato, Hitoshi Miyashita, Takayuki Harata, Toshiro Imai, Masanobu Hayashi, Shinyo Ishi, Miki Shoji, Yoshimichi Imai

**Affiliations:** a Department of Plastic and Reconstructive Surgery, Tohoku University Graduate School of Medicine, 2-1 Seiryo-cho, Aoba-ku, Sendai 980-8575, Japan; b Department of Otorhinolaryngology, Head and Neck Surgery, Tohoku University Graduate School of Medicine, 2-1 Seiryo-cho, Aoba-ku, Sendai 980-8575, Japan; c Maxillofacial Prosthetics Clinic, Tohoku University Hospital, 2-1 Seiryo-cho, Aoba-ku, Sendai 980-8575, Japan; d Department of Dentistry and Oral Surgery, Tohoku Medical and Pharmaceutical University Hospital, 1-12-1 Fukumuro, Miyagino-ku, Sendai 983-8512, Japan; e Dental Laboratories, Central Clinical Facilities, Tohoku University Hospital, 2-1 Seiryo-cho, Aoba-ku, Sendai 980-8575, Japan

**Keywords:** Mandibular reconstruction, Fibular flap, Oral intake, Denture, Teeth, Virtual surgical planning

## Abstract

**Background:**

Computer-assisted mandibular reconstruction after mandibulectomy enables accurate reconstruction of the occlusal positions between the maxilla and mandible. Understanding the remaining teeth condition is essential for sensory mastication in patients with numerous tooth loss. However, no studies have examined the dental status of the remaining mandible after computer-assisted mandibular reconstruction using a fibular flap. This study evaluated the role of residual teeth and other factors in effective acquisition of oral intake after computer-assisted mandibular reconstruction using a fibular flap.

**Patients and Methods:**

Postoperative oral intake and associated factors were retrospectively examined in 57 consecutive patients. Oral intake was assessed using the Functional Oral Intake Scale. Multivariate analysis was performed to evaluate the remaining teeth arrangement (Eichner's classification), mandibular dentures, extent of resection (Brown's classification), age, performance of glossectomy, history of radiation therapy, and computer-assisted methods.

**Results:**

Multivariate analysis revealed that Eichner's classification had a positive (p<0.001) and radiation therapy had a negative (p<0.05) impact on oral intake. The patients with dentures anchored to the remaining teeth in the occlusal support area (i.e., premolar and molar) had higher Functional Oral Intake score than those with dentures anchored to the remaining teeth in the non-occlusal support area (6.78±0.03 vs. 6.10±0.07, p<0.005).

**Conclusion:**

In computer-assisted mandibular reconstruction with accurate occlusion, the residual teeth in the occlusal support area are essential for good postoperative oral intake with dentures. During mandibulectomy, if oncologically acceptable, these teeth should be preserved, and selective placement of dental implants in the occlusal support area should be considered.

## Introduction

A vascularized free fibular flap is a common rigid material for mandibular reconstruction; however, in patients with large defects requiring multiple osteotomies of the fibula, achieving a precise mandibular outline and optimal occlusion can be challenging.^1^^,^^2^ However, in recent years, computer-assisted surgery using full-scale custom-made three-dimensional (3D) mandibular models based on computed tomography (CT) data^3^^,^^4^ and computer-aided design (CAD)/computer-aided manufacturing (CAM) techniques has significantly improved the accuracy of mandibular reconstruction.^1^^,^^5^^,^^6^ Thus, computer-assisted surgery enables accurate fibular placement in the defect and optimal occlusion of the residual mandibular teeth opposing the maxillary teeth.

Residual mandibular teeth are crucial for mastication after reconstructive patients, as are newly created dentures and implants. Additionally, residual mandibular teeth are particularly advantageous because they can be used immediately after surgery and serve as anchors for dentures from the early postoperative period. Furthermore, the residual mandible has perceptive teeth and can masticate with appropriate occlusal force due to dental root perception; however, the newly reconstructed area has no teeth or perception. Thus, residual teeth are considered to play an important role for patients after reconstructive surgery; however, no studies have examined the dental status of the remaining mandible after computer-assisted mandibular reconstruction using a fibular flap.

This study aimed to evaluate the role of residual teeth and other factors affecting oral intake after computer-assisted mandibular reconstruction using a fibular flap.

## Patients and methods

This retrospective study was conducted in accordance with the principles of the Declaration of Helsinki. All protocols were approved by the Institutional Review Board of Tohoku University Hospital (approval number: 2018-1-736). Fifty-seven patients who underwent free fibular flap reconstruction after mandibulectomy at Tohoku University Hospital between July 2013 and December 2020 were selected and reviewed. Informed consent was obtained from the patients for undergoing computer-assisted mandibular reconstruction using autologous bony free flaps and the follow-up procedures.

### Surgical technique

At our institution, the method of computer-assisted reconstruction has been in transition, with cases operated on from July 2013 to October 2016 using full-scale 3D models from 3D printers, and cases operated on after November 2016 as in-house CAD/CAM-assisted cases. Therefore, we used full-scale customized 3D models and CAD/CAM as factors for computer-assisted surgical support.

A full-scale custom-made 3D model of the mandible was created preoperatively using a 3D printer (3D Systems Projet 460 Plus; RICOH, Tokyo, Japan) based on Standard Triangulated Language data obtained from a CT scan of the facial bones. The mandibular reconstruction plate was bent according to the 3D model. During surgery, the mandibular reconstruction plate was fixed in place with intermaxillary fixation of the bite plate after mandibulectomy. An osteotomized fibula was then placed and fixed in the mandibular defect created after plate fixation.^4^

The surgical procedure with the in-house CAD/CAM support is presented below (for details, see Ohkoshi et al. 2021).^7^ The craniofacial bones and fibula were scanned using CT, and the resulting stereolithography files were imported into the CAD software (Dental Modeling Software; Toyotsu Machinery, Nagoya, Japan) to create 3D virtual models of the maxillofacial bones and fibula. A mandibular and fibula cutting guide and mandibular reconstruction model were then created based on the virtual simulation. The mandibular reconstruction plate was bent in accordance with the mandibular reconstruction model. When fixing the mandibular reconstruction plate, the remaining mandible was repositioned to the pre-excision position using a tray for fitting the mandibular reconstruction model after intermaxillary fixation.^8^^,^^9^

In cases where postoperative radiation therapy was planned and the risk of postoperative exposure of the mandibular reconstruction plate was judged to be high, miniplate fixation was performed; however, even in such cases, the tray was used during fibula placement to accurately reproduce the jaw position.

The Functional Oral Intake Scale (FOIS) score was used in this study to measure masticating and swallowing functions; the FOIS score was assessed 1-year postoperatively.^10^ The FOIS scores measured in this study were recorded for patients who wear dentures while eating, and for patients who had no dentures or did not wear them while eating due to unstable denture fixation, the scores were obtained without dentures. The patients with remaining teeth wore dentures anchored to the remaining teeth, and edentulous patients wore dentures on the remaining alveolar and reconstructed sites (without dental implants). All dentures were worn on the residual and reconstructed areas of the mandible. All dentures were fabricated approximately 6 months postoperatively to match the morphology of the remaining teeth and soft tissues covering the fibula. No patients had new dental implants placed in the mandibular or maxilla during the study period.

Eichner's classification, based on occlusal contacts in the posterior region, was used to arrange the remaining teeth. The region was divided into 4 occlusal support areas, and occlusal contacts were assessed without dentures.^11^ The 3 groups (A, B, and C) with 4 subgroups indicated the number of occlusal support areas as follows: A, 4 support areas; B1, 3 support areas; B2, 2 support areas; B3, one support area; B4, no support area with anterior occlusal contact; and C, no occlusal contact. In this study, there were no cases corresponding to groups A and B1 due to mandibular resection, and the cases were classified into 4 categories: B2, B3, B4, and C. Furthermore, when examining the relationship between the denture and remaining teeth, we divided the 4 categories into 2 groups: B2 and B3 with occlusal support areas and B4 and C with non-occlusal support areas.

Because postoperative oral intake depends on the extent of mandibular resection,^12^ the extent of mandibular defects was divided into 2 groups using Brown's classification: I and II, lateral defect groups and III and IV, anterior defect groups.^13^ The patients who underwent condylar resection were included in the lateral defect group.

Finally, the variables affecting the FOIS score included age, Eichner's classification, whether a denture was worn while eating, Brown's classification, use of a full-scale 3D model or CAD/CAM as a surgical support method, whether glossectomy was performed, and whether pre- or postoperative radiation therapy was used. We included cases with preoperative radiotherapy that had previously been irradiated to the mandible for oropharyngeal cancer or maxillary cancer. However, we also included cases with postoperative radiotherapy that had received radiation for positive margins after mandibulectomy or extracapsular invasion of lymph nodes. Cases with local recurrence were excluded from the analysis.

### Statistical analysis

For univariate analysis, the Mann–Whitney U test was used to compare differences between the 2 groups on ordinal scales, and Spearman's rank correlation coefficient was used to test for correlation. For the multivariate analysis, we used multiple ordinal logistic analyses. We transformed Eichner's classification into a dummy variable and confirmed that it was an ordinal scale based on its correlation with the FOIS. Forced entry methods were used to select explanatory variables, and the final variable selection was determined from the model's goodness of fit by calculating multicollinearity and performing likelihood ratio tests. Statistical significance was set at p<0.05. All statistical tests were two-tailed. All statistical analyses were performed using R for Mac version 3.5.0 (R Foundation for Statistical Computing, Vienna, Austria).

## Results

A total of 57 consecutive patients were included in the study. Because the FOIS score was measured at 1 year postoperatively, the records of 51 patients were analyzed after excluding 3 cases in which the reconstructive plate was removed due to complications within the first postoperative year and 3 cases of local recurrence. All patients (35 males and 16 females; median age 65.5 years [range, 30–76 years]) underwent mandibular reconstruction using a vascularized fibular flap after mandibulectomy. Table 1 details the patient characteristics.

**Table 1:** Characteristics of the patients.

Characteristic	Patients (N=51)
Sex	
Male	35
Female	16
Primary disease	
Lower gingival cancer	38
Buccal mucosal cancer	3
Floor of mouth cancer	3
Osteosarcoma	1
Submandibular grand cancer	1
Ameloblastoma	3
Osteoradionecrosis	2
Neck dissection	
Bilateral neck dissection	9
Hemi neck dissection	38
No dissection	4

The variables, number of patients, mean FOIS score, and results of the univariate analysis are shown in Table 2. A significant positive correlation was found between Eichner's classification (C, B4, B3, B2) and FOIS scores (Spearman's rank correlation coefficient: 0.655, p<0.001). The FOIS score of the group with dentures was significantly higher than that of the group without dentures (p<0.005). The FOIS score of the lateral defect group (classes I and II) was significantly higher than that of the anterior defect group (classes III and IV) (p<0.005). There was no significant difference between the computer-assisted methods (p=0.158). There were 10 cases of glossectomy, of which 6 were partial glossectomies, 1 was a hemiglossectomy, and 3 were subtotal glossectomies. Patients who underwent a hemiglossectomy or subtotal glossectomy required reconstruction with another free flap and a fibular flap. The FOIS score of the non-glossectomy group was significantly higher than that of the glossectomy group (p<0.05). The FOIS score of the non-radiation therapy group was significantly higher than that of the radiation therapy group (p<0.05).

**Table 2:** Results of univariate analysis to assess factors contributing to postoperative oral intake function.

Variable	Patients (N=51)	Mean (±SE) FOIS score	p-value
Age (years)			
≤65	25	6.04±0.057	0.84
>65	26	6.42±0.029	
Eichner's classification			
B2	23	6.83±0.021	<0.001***
B3	8	6.75±0.083	
B4	4	6.25±0.207	
C	16	5.13±0.079	
Denture wear			
Yes	37	6.60±0.018	<0.005**
No	14	5.29±0.109	
Brown's classification			
Ⅰ or Ⅱ	36	6.53±0.025	<0.005**
Ⅲ or Ⅳ	15	5.53±0.091	
Surgical support method			
Full-scale 3D model	19	5.95±0.071	0.158
CAD/CAM	32	6.41±0.030	
Glossectomy			
Yes	10	5.70±0.110	<0.05*
No	41	6.37±0.027	
Radiation therapy			
Yes	16	5.75±0.090	<0.05*
No	35	6.46±0.026	

FOIS, functional oral intake scale; SE, standard error; CAD/CAM, computer-aided design/computer-aided manufacturing; 3D, three-dimensional.

Mann–Whitney U test, *p<0.05 **p<0.005 ***p<0.001.

To further investigate the relationship between denture wearing status, Eichner's classification, and oral intake function, an additional study was conducted (Table 3). Patients with remaining teeth in the occlusal support area (i.e., Eichner's classification B2 and B3; 87%) were more likely to wear dentures than those with remaining teeth in the non-occlusal support area (i.e., Eichner's classification B4 and C; 50%). In a study of patients with dentures, the FOIS score of the patients with denture anchored to the remaining teeth on the occlusal support area was significantly higher than that of those with denture anchored to the remaining teeth on the non-occlusal support area (6.78 ± 0.03 vs. 6.10 ± 0.07, Mann–Whitney U test, p<0.005).

**Table 3:** The relationship between Eichner's classification and FOIS score in denture wearing patients.

	Eichner's classification		p-value
	B2 and B3	B4 and C	
**Patients with Denture wearing**	27	10	
**Mean ± SE FOIS score**	6.78 ± 0.03	6.10 ± 0.07	<0.005**

FOIS, functional oral intake scale; SE, standard error.

Mann–Whitney U test, *p<0.05 **p<0.005 ***p<0.001.

In the multivariate analysis, a model containing 4 explanatory variables (Eichner's classification, denture wearing status, CAD/CAM, and radiation therapy) was selected based on the model's goodness of fit (Table 4).

**Table 4:** Multiple ordinal logistic regression analysis to assess the independent factors related to postoperative oral intake function.

Variable	Odds ratio	95% CI	p-value
Surgical support method	2.70	0.74-10.30	0.14
Denture wearing	4.50	0.97-21.40	0.055
Eichner's classification	2.92	1.71-5.43	<0.001⁎⁎⁎
Radiation therapy	0.21	0.05-0.80	<0.05*

CI, confidence interval.

⁎⁎⁎ p<0.001.

⁎⁎ p<0.05.

Among these factors, Eichner's classification had a positive impact (p<0.001) and radiation therapy had a negative impact (p<0.05) on oral intake function. In contrast, the computer-assisted reconstruction method did not contribute to the postoperative FOIS score (p=0.14). Similarly, denture wearing was slightly below the significance level, but to a lesser extent (p=0.055).

### Case 1

**Figure 1:**
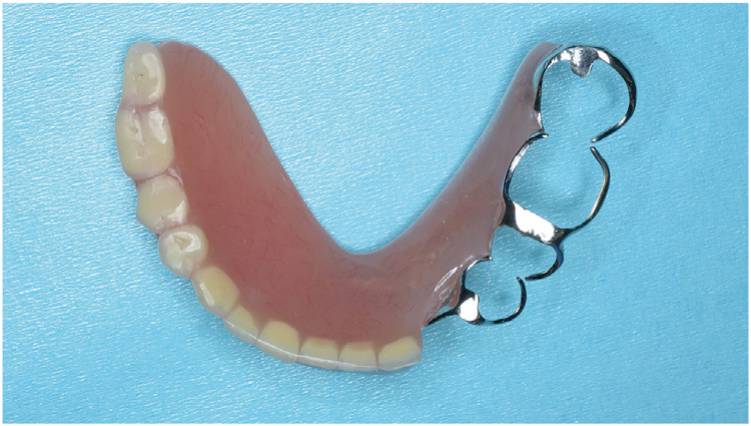
A denture anchored to the remaining premolar and molar in the occlusal support area is created.

**Figure 2:**
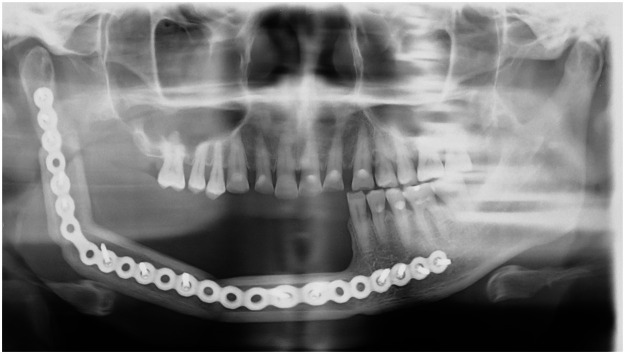
Orthopantomography at 1 year postoperatively. The occlusion between the residual mandibular teeth and the maxillary teeth is optimal.

**Figure 3:**
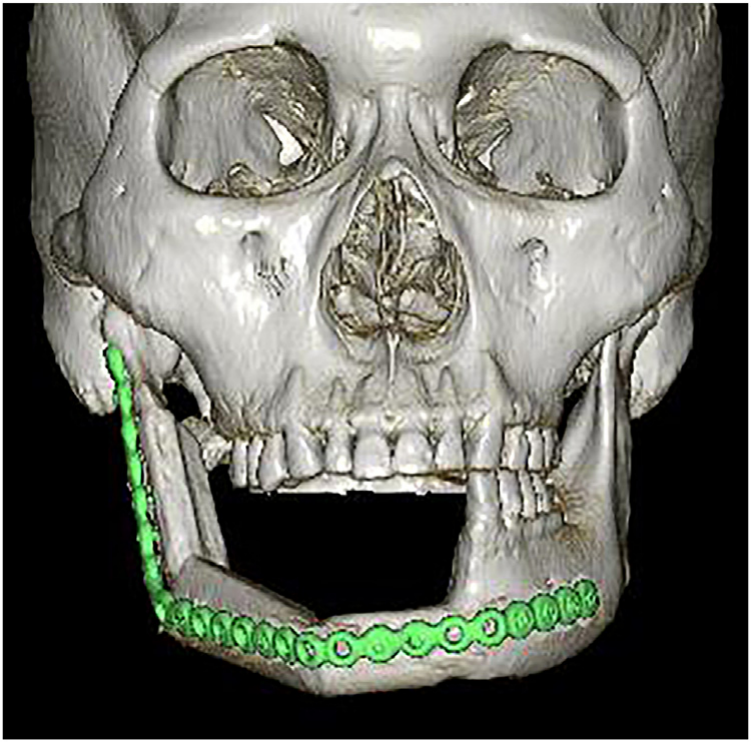
3D-CT at 1 year postoperatively. The reconstructed mandibular morphology is good. 3D-CT, three-dimensional computed tomography.

**Figure 4:**
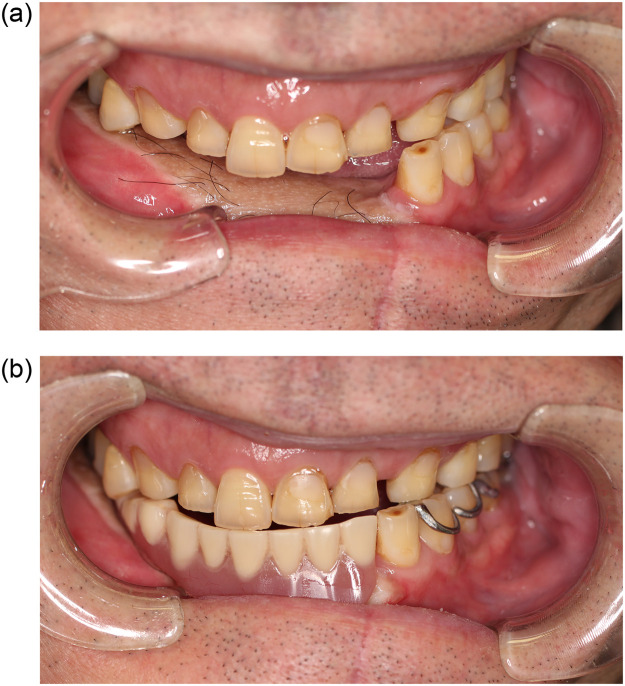
The state of occlusion of the remaining teeth without denture (a) and with denture (b).

A 58-year-old male patient with gingival cancer (cT4bN2bM0) underwent hemimandibulectomy (Brown's classification Ⅱ) and right neck dissection. The mandible was reconstructed using a free fibular flap with in-house CAD/CAM support. His remaining teeth were present in the 2 occlusal support areas (Eichner's classification B2). Six months postoperatively, a denture anchored to the remaining teeth was created (Figure 1). At 1 year postoperatively, the occlusion and reconstructed mandibular morphology were good (Figure 2, Figure 3). He wore the denture while eating and consumed a regular diet with no restrictions (FOIS score 7, Figure 4).

### Case 2

**Figure 5:**
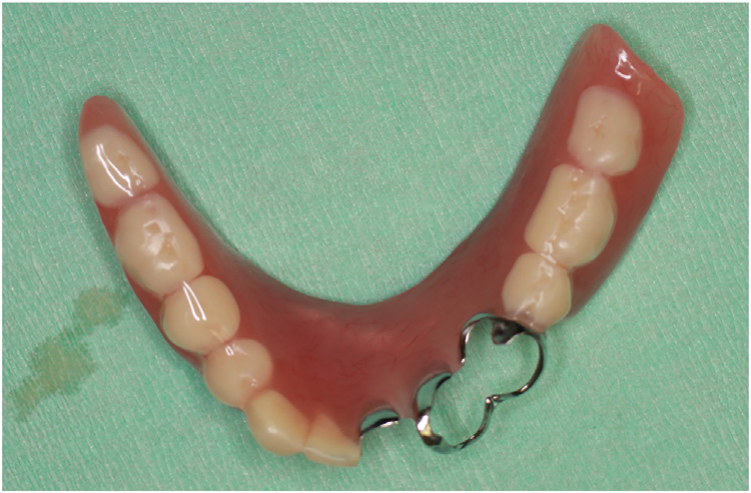
A denture anchored to the remaining teeth in a non-occlusal support area is created.

**Figure 6:**
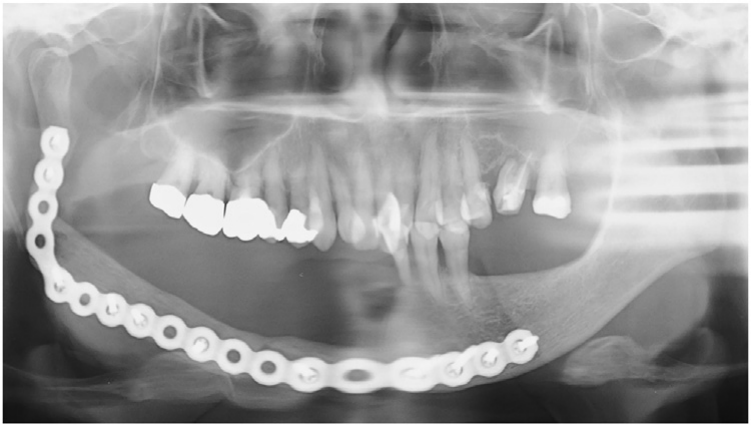
Orthopantomography at 1 year postoperatively. The occlusion between the residual mandibular teeth and the maxillary teeth is optimal.

**Figure 7:**
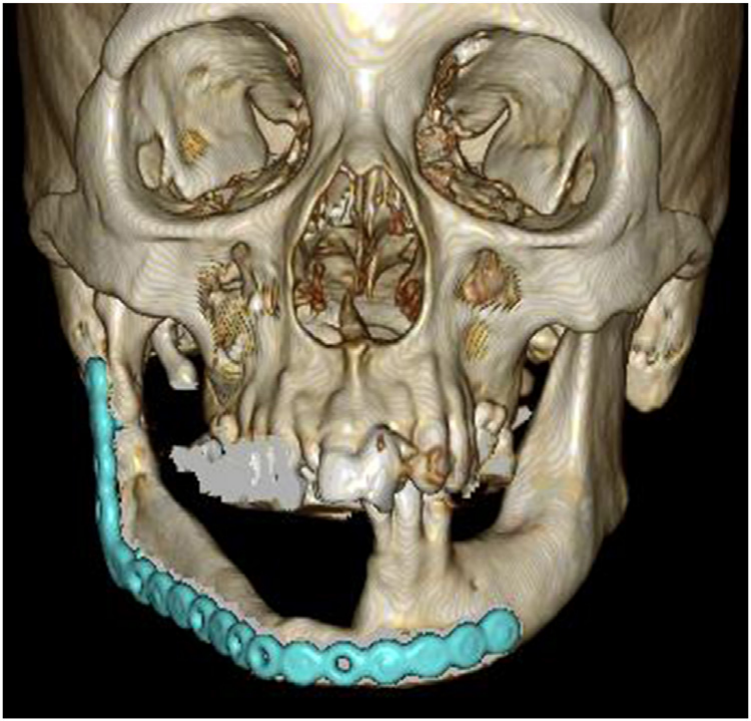
3D-CT at 1 year postoperatively. The reconstructed mandibular morphology is good. 3D-CT, three-dimensional computed tomography.

**Figure 8:**
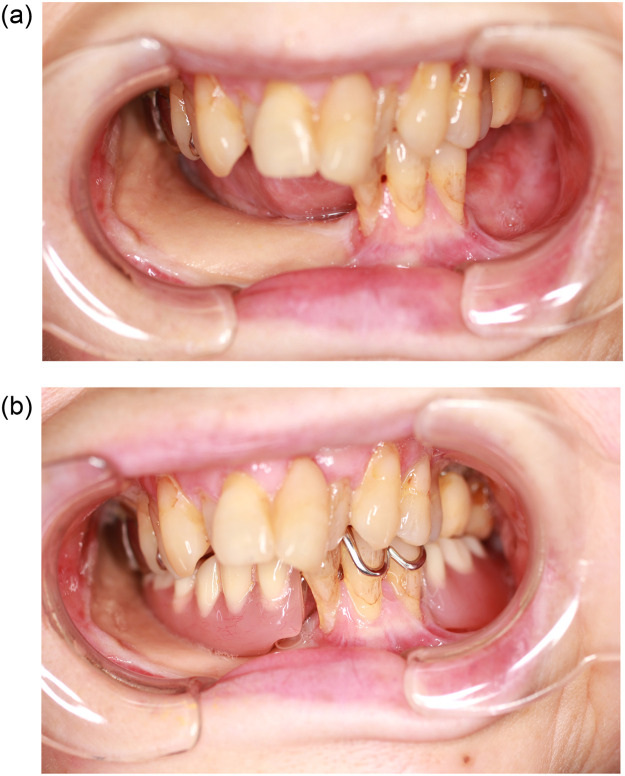
The state of occlusion of the remaining teeth without denture (a) and with denture (b).

A 68-year-old female patient with gingival cancer (cT4aN1M0) underwent hemimandibulectomy (Brown's classification Ⅱ) and right neck dissection. The mandible was reconstructed using a free fibular flap with in-house CAD/CAM support. Her remaining teeth were not present in the occlusal support area and had anterior occlusal contact (Eichner's classification B4). Six months postoperatively, a denture anchored to the remaining teeth was created (Figure 5). At 1 year postoperatively, both occlusion and reconstructed mandibular morphology were satisfactory (Figure 6, Figure 7). She wore the denture while eating; however, the denture was unstable (Figure 8). The denture was used not for direct mastication but to prevent the movement of the food masses to the buccal side during chewing and swallowing. Thus, she was unable to chew hard foods (FOIS score 6).

## Discussion

Tsuchiya et al. reported that the existence of opposing teeth contributes to postoperative eating function in patients who undergo mandibular reconstruction; however, the study was conducted among patients who did not undergone computer-assisted reconstructive surgery.^14^ To our knowledge, this is the first study to focus on the role of residual teeth in the evaluation of eating function after computer-assisted mandibular reconstruction. Computer-assisted surgery has rapidly evolved in recent years, enabling accurate reconstruction. However, previous reports have discussed reconstruction methods such as tissue grafting, fixation of the reconstruction plate,^5^^,^^15^, ^16^, ^17^, ^18^ and dental implant placement^19^ and their accuracy for the defect sites. We believe that digital technology contributes more to the accuracy of occlusion of the remaining teeth, particularly to the accuracy of the reconstructed site with free tissue transfer.

This study demonstrated that residual teeth were the most influential factor affecting oral intake in computer-assisted surgery (Table 1, Table 2). The oral intake function was best when the teeth remained in the occlusal support area, followed by the remaining teeth in the anterior region and the edentulous mandible (Table 1). This provides guidance for selecting the appropriate postoperative diet according to Eichner's classification. There are 2 reasons why the oral intake function is better when the teeth remain in the occlusal support area than when they remain in the non-occlusal support area. The first is the difference in the inherent nature of the remaining teeth: molars and premolars originally have more masticatory function than the incisors, lateral incisors, and canines. The second reason is the usefulness of dentures as a source of fixation. In fact, the denture wearing rate in the occlusal support area was higher than that in the non-occlusal support area (27/31 vs. 10/20 patients, Table 3).

In a detailed survey of denture attachment after mandibular reconstruction in non-computer-assisted surgeries, Mochizuki et al. reported that patients with Eichner's classifications B and C had improved masticatory function after placing the dentures.^20^ Our results also showed that patients with dentures had better oral intake than those without dentures (Table 2), and that in a study in which patients wearing denture were selected, the occlusal support area were better suited to fix dentures than the non-occlusal support area with respect to oral intake (6.78±0.03 vs. 6.10±0.07, Mann–Whitney U test, p<0.005, Table 3). This suggests that for patients who have lost many teeth after reconstruction, the placement of new dental implants in the occlusal support area may be particularly useful for improving masticatory function. In addition, if oncologically acceptable, preservation of molars or premolars should be considered during mandibulectomy for cancer treatment to ensure adequate postoperative oral intake.

Multivariate analysis showed that a history of radiation therapy had an adverse effect on oral intake, which is consistent with previous reports.^21^^,^^22^ One year postoperatively, the postradiotherapy patient already had a denture made. At 1 year postoperatively, we considered the patient's oral intake function to be stabilized because it has been more than 6 months since the patient received radiotherapy at our institution. Because our institution does not fabricate implants in the radiation field, we do not expect these patients to have improved feeding function in the future with implants. The extent of mandibular defects affects the eating function after mandibular reconstruction.^12^ Brown's classification was included as a factor of consideration in this study, but was excluded during the analysis. This is because Eichner's classification, which is confounded by Brown's classification, was found to have a statistically greater impact on oral intake function than Brown's classification. Although it is known that anterior resection of the mandible makes laryngeal elevation impossible and causes dysphagia, at our institution, anterior mandibular defects are treated in conjunction with laryngeal elevation to address the loss of function. All 15 patients with Brown's classifications III and IV included in this study underwent laryngeal elevation as an adjunctive procedure during reconstructive surgery. Notably, a confounding factor exists between the extent of mandibular resection and oral intake function, namely, the presence or absence of adjuvant surgery.

Finally, multivariate analysis showed that glossectomy did not affect oral intake function. It is possible that the sample size was too small to be detected; however, it can be interpreted that appropriate tongue reconstruction can prevent some loss of oral intake function. Regarding dental rehabilitation associated with mandibular reconstruction, it is important to remember the significance of tongue function in mastication. Surgeons may consider using free flaps to replace the missing portions of the anterior tongue if indicated.^23^ This study has a limitation that it was a single-center, small-sample, retrospective study. A multicenter cooperative study with a larger sample size is needed to examine the role of factors other than the residual teeth in postoperative oral intake.

In conclusion, in computer-assisted mandibular reconstruction with accurate occlusion, the remaining teeth in the residual mandible play an important role in maintaining the postoperative oral intake. Anchoring dentures to the remaining premolars and molars in the occlusal support area is essential for postoperative oral intake. If oncologically acceptable, during mandibulectomy, these teeth should be preserved. Postoperatively, regarding the reconstructed site, selective placement of new dental implants in the occlusal support area should be considered.

## Conflict of interest

None.
